# What Can Neural Activity Tell Us About Cognitive Resources in Aging?

**DOI:** 10.3389/fpsyg.2021.753423

**Published:** 2021-10-18

**Authors:** Chiara F. Tagliabue, Veronica Mazza

**Affiliations:** Center for Mind/Brain Sciences (CIMeC) – University of Trento, Rovereto, Italy

**Keywords:** cognitive aging, cognitive resources, working memory, interindividual variability, aging trajectories, neural correlates, age-related brain activity change

## Abstract

A reduction in cognitive resources has been originally proposed to account for age-related decrements in several cognitive domains. According to this view, aging limits the pool of available cognitive supplies: Compared to younger adults, elderly exhaust the resources more rapidly as task difficulty increases, hence a dramatic performance drop. Neurophysiological indexes (e.g., BOLD response and EEG activity) may be instrumental to quantify the amount of such cognitive resources in the brain and to pinpoint the stage of stimulus processing where the decrement in age-related resources is evident. However, as we discuss in this mini-review, the most recent studies on the neurophysiological markers of age-related changes lack a consistent coupling between neural and behavioral effects, which casts doubt on the advantage of measuring neural indexes to study resource deployment in aging. For instance, in the working memory (WM) domain, recent cross-sectional studies found varying patterns of concurrent age-related brain activity, ranging from equivalent to reduced and increased activations of old with respect to younger adults. In an attempt to reconcile these seemingly inconsistent findings of brain-behavior coupling, we focus on the contribution of confounding sources of variability and propose ways to control for them. Finally, we suggest an alternative perspective to explain age-related effects that implies a qualitative (instead of or along with a quantitative) difference in the deployment of cognitive resources in aging.

## Age-Related Reduction in Processing Resources

A marked decline across several cognitive domains is a common feature of aging (Hedden and Gabrieli, 2004). To account for this performance reduction, the *processing resources* framework (Craik and Byrd, 1982; Salthouse, 1988, 1990) posits that aging implies a decline in the amount of available cognitive resources, in that older individuals exhaust them more rapidly than younger adults. According to this account, the performance drop, measured as task requests increase, occurs because old individuals consume processing resources already at low levels of cognitive demand. The age-related changes in working memory (WM) capacity, a hallmark of cognitive aging (Myerson et al., 2003), nicely fit with this perspective. Indeed, in simple short-term memory tasks (mainly tapping on storage capacity, e.g., forward digit span), old adults are negligibly compromised. However, compared to young, they become impaired in WM tasks requiring additional cognitive processing (namely, concurrent storage and manipulation of items, e.g., backward or complex digit span; see Bopp and Verhaeghen, 2005, for a review). These effects may be interpreted within the processing resources framework: The reduced pool of cognitive resources in aging is sufficient for the elderly to efficiently perform in simple tasks (e.g., short-term memory tasks), but not when they have to face higher cognitive demands (e.g., in WM tasks), and thus, a greater performance decrement is visible. An interesting approach has been proposed to substantiate the hypothesis of age-related reduction in processing resources (thought to be responsible for the observed behavioral deficits): measuring the neural activity (e.g., BOLD response and M/EEG activity) during the execution of various tasks, and isolating specific indexes that mirror the hypothesized resource decrement (e.g., McEvoy et al., 2001; Mattay et al., 2006). This approach is beneficial for at least two reasons. First, it provides an additional (cerebral) measure to quantify the amount of available resources in the elderly; second, it individuates the specific neural and functional mechanism where age-related differences in processing deployment originate. As Salthouse (1988) originally suggested, the candidate neural index of cognitive resources should positively correlate with cognitive performance (i.e., the greater the cerebral recruitment, the better the performance) and negatively correlate with age (i.e., the older the participant, the more reduced the cerebral activity). However, finding a neural measure that satisfies these requirements has proven to be difficult in the field of aging research. Indeed, results obtained from cross-sectional studies highlight that a consistent coupling between neural and cognitive modulations is currently lacking, which complicates predictions on how the neural markers of cognitive resource deployment should be modulated by age. A review of the most recent (in the last 5years) imaging studies (fMRI and M/EEG) on WM provides substantial examples of these non-unidirectional patterns of age-related brain activity. Indeed, WM has been proposed as an ideal domain to test for the presence of any age-related variation in cognitive resources, since it is defined by a limited capacity and is relevant to other cognitive domains (Salthouse, 1990). In the next section, we will briefly illustrate some of the most recent results (note, however, that similar conclusions can be drawn when also considering articles published earlier than 2016). As we will describe, linking brain and cognition in an attempt to quantify the amount of available processing resources in aging is far from being a straightforward research approach.

## Neural Indexes Underlying Cognitive Resource Deployment in Aging

fMRI and M/EEG studies investigating WM in young and older adults have used various tasks (e.g., verbal and visuo-spatial n-back, delayed match-to-sample, Corsi-Block Tapping, and Sternberg paradigm; see Table 1 for further details on recent studies). Across these paradigms, elderly usually exhibit a reduction of WM capacity compared to young adults. However, such decrements in performance are mirrored by different patterns of brain activity.

**Table 1:** Neuroimaging studies comparing young and older adults in WM tasks and published from 2016 onwards.

Article	Methodology	Task
Archer et al., 2018	fMRI	Spatial Addition Task
Bauer et al., 2018	fMRI	Modified version of Corsi-Block-Tapping test
Crowell et al., 2020	fMRI	Verbal WM manipulation task of consonant strings
Gallen et al., 2016	fMRI	Visual n-back task
Heinzel et al., 2017	fMRI	Numerical n-back task
Höller-Wallscheid et al., 2017	fMRI	Verbal, spatial and object-based delayed match-to-sample task
Jamadar, 2020	fMRI	Visuo-spatial sequence paradigm
Kennedy et al., 2017	fMRI	Numerical n-back task
Qin and Basak, 2020	fMRI	Modified 2n-back task with colored digits
Rieck et al., 2021	fMRI	Verbal n-back task
Vellage et al., 2016	fMRI	Visuo-spatial delayed match-to-sample task
Billig et al., 2020	EEG	Visual delayed match-to-sample task
Hou et al., 2018	EEG	Verbal n-back task
Lubitz et al., 2017	EEG	Spatial n-back task
Morrison et al., 2019	EEG	Numerical n-back task
Sghirripa et al., 2021	EEG	Sternberg task
Tagliabue et al., 2019, 2020	EEG	Visuo-spatial delayed match-to-sample task
Tran et al., 2016	EEG	Visuo-spatial delayed match-to-sample task
Leenders et al., 2018	MEG	Visuo-spatial delayed match-to-sample task
Proskovec et al., 2016	MEG	Sternberg task

*To simplify result comparison, articles using dual-task or dual-task-like paradigms are not included*.

Recent fMRI studies (Gallen et al., 2016; Heinzel et al., 2017; Kennedy et al., 2017; Archer et al., 2018; Bauer et al., 2018; Jamadar, 2020; Qin and Basak, 2020) show that the behavioral decrease in WM capacity of old adults is coupled with equal, increased, or reduced brain activation relative to younger adults. Moreover, different brain regions (or even different portions within the same region) show opposite patterns of age-related activity: While some of them are underrecruited, others appear overactive with respect to young. For instance, age-related decrement in WM performance can be accompanied by a reduced activation of task-related areas – middle frontal gyrus and bilateral precunei – together with increased activation of task-unrelated regions – cuneus, temporal gyrus, and cerebellum (Archer et al., 2018). In addition, at lower levels of task demand elderly can exhibit larger activations in frontal and parietal areas (Heinzel et al., 2017), but also reduced BOLD activity in frontal and temporal regions, with concurrent larger activation in the bilateral cuneus (Jamadar, 2020). Likewise, connectivity measures for easy task conditions indicate increased connectivity between lateral frontal areas and other networks with increasing age (Gallen et al., 2016), but no difference in connectivity strength between frontal and parietal regions (Heinzel et al., 2017).

Similar findings are observed when M/EEG studies are considered (Schwarzkopp et al., 2016; Tran et al., 2016; Lubitz et al., 2017; Morrison et al., 2019; Tagliabue et al., 2019, 2020): Taken together, the findings indicate that components reflecting attentional engagement and maintenance in WM may be enhanced, reduced or similar between age groups, even in the presence of marked behavioral differences. For instance, some EEG studies found decrements in older adults’ WM with a concurrent less pronounced (Lubitz et al., 2017) or enhanced fronto-central P200 (Morrison et al., 2019), an ERP component reflecting deployment of attentional resources. Additionally, when individuals are presented with different memory loads, older adults might show either similar (Schwarzkopp et al., 2016; Tran et al., 2016) or reduced (Tagliabue et al., 2019, 2020) load-related modulations of the CDA, an ERP response indexing the amount of items maintained in the WM short-term storage.

To summarize, recent findings on aging highlight an apparent lack of a unidirectional coupling between brain and behavioral outcomes. The absence of a consistent brain-behavior pattern ultimately questions the possibility of formulating testable hypotheses on age-related effects at the neural level and, more generally, whether we can reliably interpret neural activity (being it BOLD signals, ERPs, neural oscillations, or functional connectivity) to infer the amount of deployed cognitive resources in aging. In the next section, we will consider potential sources of variability accounting for these different effects.

## Potential Sources of Variability Accounting For Different Age-Related Patterns of Resource Deployment in Working Memory

At least two sources of variability can account for the different brain-behavior associations in the WM domain. First, as previously mentioned, various cognitive tasks have been used to test WM functioning. Even if meant to assess the same cognitive function, different experimental paradigms can yield different results for at least two (partially related) main reasons. Different tasks might selectively engage different cognitive subcomponents (and their respective neural substrates), depending on their experimental structure (e.g., delayed match-to-sample paradigms tax more information maintenance and retrieval abilities, whereas n-back tasks rely more on information updating; see Daniel et al., 2016; Yaple et al., 2019) and type of material (verbal and visuo-spatial). Consequently, some tasks can intrinsically be more difficult than others. For instance, regarding the experimental structure, the overall accuracy in an n-back task is lower than in a Sternberg test (see Heinzel et al., 2016). With reference to stimulus material, elderly are usually more impaired with visuo-spatial than verbal items (Jenkins et al., 2000).

The second source of variability that may account for non-unidirectional age-related patterns is interindividual variability. Interindividual variability is prominent in aging (Lindenberger and von Oertzen, 2006) and may lead to optimal or less successful aging trajectories (Reuter-Lorenz and Park, 2010; Reuter-Lorenz and Park, 2014; Cabeza et al., 2018). Indeed, in some studies, the sample of older adults might include high-performing participants that can bias the group average performance toward one direction (and vice versa in the case of low-performing elderly). This heterogeneity in aging trajectories is largely due to age-related changes at multiple levels of neurobiological function and structure (Raz and Daugherty, 2018), in interaction with environmental factors (Daffner et al., 2011). Thus, interindividual variability in aging may underlie differences in the expression of brain activations (Cabeza et al., 2018). Specifically, preserving a good cognitive level at old age could be reflected by either a youth-like functioning brain (i.e., no age-related differences in brain activity; e.g. Pudas et al., 2013), an overactivation of some areas and/or supplementary engagement of an alternative set of brain networks (see Spreng et al., 2010 for a review) that might act as compensatory mechanisms to support the behavioral performance.

In the next section, we will consider possible solutions to minimize task-related variability and to better operationalize individual differences. Indeed, when sources of variability are (at least partially) accounted for, a more consistent pattern of age-related neural effects emerges, that can be more easily interpreted in the framework of cognitive resource deployment with a life span perspective.

## How Can Cognitive Resources in Aging Be More Reliably Measured Through Neural Indexes?

The use of various experimental paradigms to address the same cognitive function and individual differences are two major sources of variability that could explain the heterogeneity of findings in aging research. In particular, since individual differences are typically more prominent in older than young adults, they have been suggested to bias (e.g., by under- or over-estimating) the age-related differences observed in cross-sectional studies, where aging is implicitly treated as a uniform process (Schneider-Garces et al., 2010).

In an attempt to reduce the joint influence of task-related and interindividual variability, some studies (e.g., Höller-Wallscheid et al., 2017) have exploited procedures to equate the subjectively perceived difficulty of a specific task between age cohorts (and, in turn, across participants). These studies often apply titration procedures to match the difficulty level between young and older adults, namely, a stimulation “threshold” yielding the same accuracy value is chosen for each individual. WM studies using these procedures often find that elderly exhibit equal or increased neural activity (with reference to the compensation mechanism previously discussed) or similar load-related modulations (but see Billig et al., 2020). Indeed, recent fMRI studies with no age-related difference in accuracy found a similar modulation as a function of task demands in frontal and parietal areas between young and old adulthood (Höller-Wallscheid et al., 2017; Crowell et al., 2020), recruitment of a more extended network of areas (Vellage et al., 2016), and increased between-networks integration with increasing difficulty in the elderly (Crowell et al., 2020). In addition, M/EEG aging studies with individually titrated difficulty levels (Leenders et al., 2018) or no absolute difference in performance between age groups (Proskovec et al., 2016; Sghirripa et al., 2021) revealed that elderly showed greater increase in cortical excitability (as indexed by greater alpha power decrease; see Rihs et al., 2009) in both hemispheres (Leenders et al., 2018; Sghirripa et al., 2021) with respect to young participants (in which larger cortical excitability was instead specific to the hemisphere primarily processing targets, i.e., the contralateral hemisphere), or greater oscillatory activity in the alpha and beta bands in additional homologous frontal and parieto-occipital regions (Proskovec et al., 2016).

However, matching task difficulty between groups (likely selecting easier task conditions for the elderly) cancels out baseline differences in performance between age cohorts and (only) reveals (potential) age-related modulations of neural activity to attain the same accuracy level. In other words, this approach proves to be useful when the research focus is on within-subject effects (e.g., in the case of individual gains in training procedures), rather than on between-groups differences. Indeed, when difficulty-matching procedures are adopted, what remains to be explained is why older adults are deficient in their WM capacity (see Ramscar et al., 2014) from the very beginning (i.e., why they perceive the same subjective difficulty of younger adults at easier task levels).

When the research focus is on the comparison between different ages, two approaches can be adopted to overcome some of the limitations imposed by cross-sectional studies previously described. On one hand, dividing individuals (both young and old) in high and low performers may offer a less spurious estimate of age-related neural changes in the utilization of cognitive resources. For instance, in an EEG study by Daffner et al. (2011), low and high performers similarly allocated processing resources with increasing difficulty, regardless of age (see also Lubitz et al., 2017 and Morrison et al., 2019 for more recent EEG studies). Similarly, an fMRI study of Nagel et al. (2009) showed that, when considered altogether, elderly exhibited compromised brain responsivity compared to younger adults. Interestingly, when participants were instead split in high and low performers, the neural pattern of high-performing older adults resembled those of low-performing, equally accurate younger adults (see also Heinzel et al., 2017, Bauer et al., 2018 and Vaqué-Alcázar et al., 2020 for more recent fMRI studies).

A second approach to account for interindividual variability and overcome the drawbacks of cross-sectional studies consists of longitudinal investigations. Indeed, results obtained from cross-sectional studies might be biased by cohort effects related to preexisting generational differences (e.g., educational attainment; see Archer et al., 2018) that can “anticipate” age-related decrements (Rönnlund et al., 2005). Longitudinal studies allow researchers to (partially) isolate the effects due to aging from those linked to other experience-related variables (e.g., historical/social background). Notably, some discrepancies between cross-sectional and longitudinal studies have been found also in neural results. For instance, several cross-sectional studies documented over-recruitment of prefrontal areas in old compared to younger adults (e.g., Rajah and D’Esposito, 2005; Davis et al., 2008). However, some longitudinal studies (Nyberg et al., 2010; Rieckmann et al., 2017) reported an age-related reduction in frontal activity. More specifically, older adults defined as decliners (i.e., individuals with WM performance decline across time, as opposed to so-called maintainers) showed decreased recruitment of the prefrontal cortex (Rieckmann et al., 2017; Vaqué-Alcázar et al., 2020, 2021). To reduce the confound of cohort effects, it might be worth contemplating the administration of routine assessment of cognitive functions throughout an individual’s life span.

## Concluding Remarks

In a framework arguing for a reduction of processing resources in aging (Craik and Byrd, 1982; Salthouse, 1988, 1990), recent neuroimaging evidence in the domain of WM has not conveyed a unidirectional coupling between behavioral and neural data. However, apparent discrepancies can be reconciled if (at least) two sources of variability are controlled for, namely, task-related and interindividual differences. Indeed, when these factors are considered, two consistent findings emerge as: (1) elderly exhibit similar or augmented neural activity with respect to younger adults and (2) older low performers or longitudinal decliners engage task-related areas to a lesser extent than their more cognitively fit peers.

**Figure 1:**
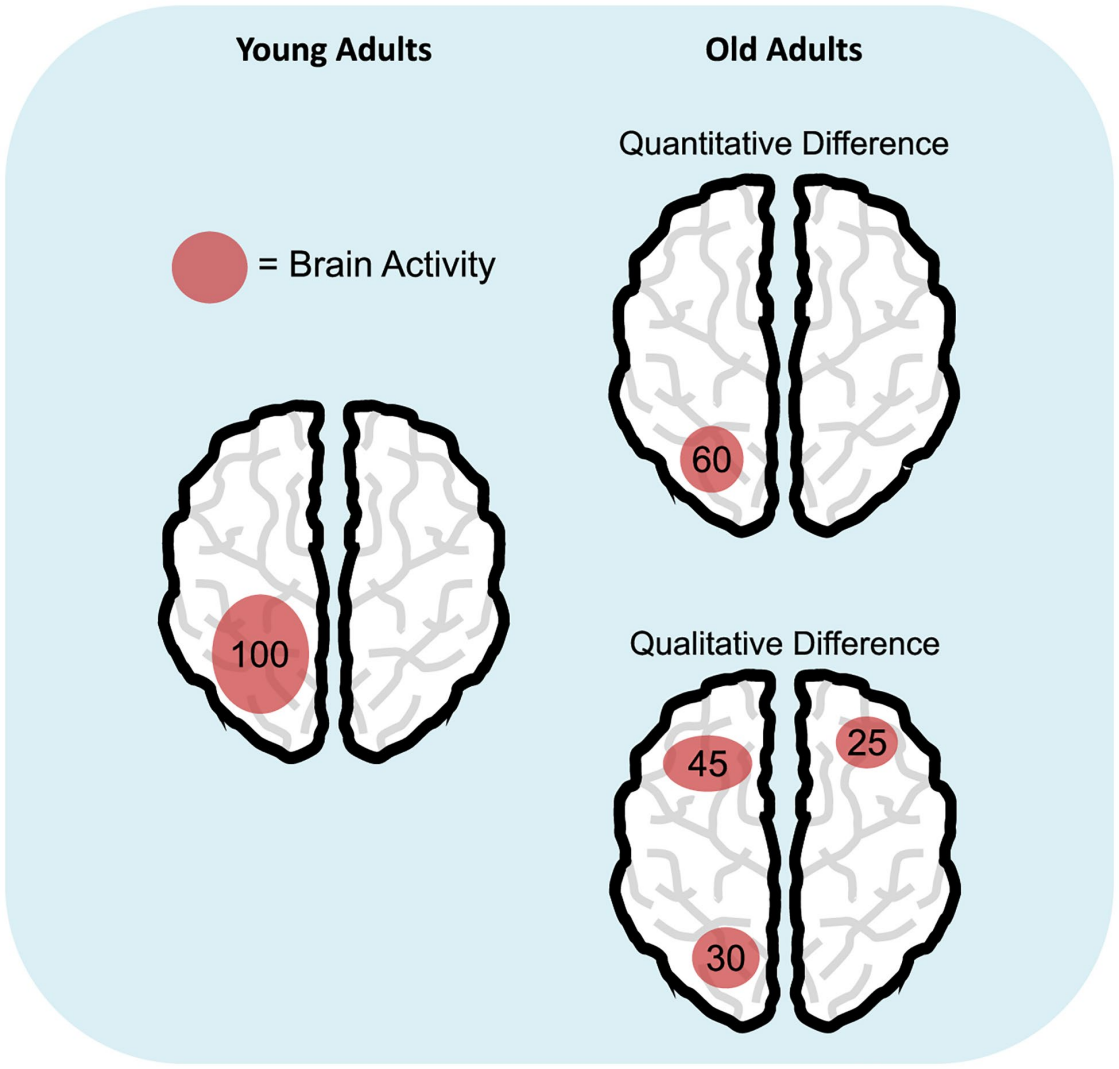
Age-related differences in cognitive resource allocation. The schematic representations illustrate the hypothesis of quantitative (i.e., reduction; top right panel) versus qualitative difference (bottom right panel) in the deployment of cognitive resources in aging, compared to young adulthood (left panel). Numbers represent a hypothetical amount of engaged cognitive resources and how they are distributed during a specific task.

Taken together, results on age-related differences in brain activity prompt for a deeper understanding of these effects, especially in the case of neural over-recruitment in the elderly, which would ideally challenge the view of reduced processing resources in aging (Salthouse, 1988). In this respect, we suggest to enlarge the hypothesis space: Rather than having a *limited* pool of resources as originally postulated (Craik and Byrd, 1982; Salthouse, 1988, 1990), older individuals may use them in a *different* (sometimes less efficient) way compared to young adults. This interpretation would imply a shift from the original view that sees aging as a (quantitative) reduction of processing resources to a novel viewpoint considering a qualitative change, not necessarily a reduction, in resource allocation (Figure 1). Several pieces of evidence support this latter perspective. First, it is well documented that aging is characterized by increased susceptibility to distraction (e.g., Hasher and Zacks, 1988; Gazzaley et al., 2008) and broader attentional focusing (Greenwood and Parasuraman, 1999, 2004). These deficits are responsible for the inadvertent processing of irrelevant material, and this may result in the typical age-related WM capacity reduction (e.g., Jost et al., 2011; Tagliabue et al., 2019). Since WM storage has a limited capacity (Cowan, 2010), WM may become deficient because old adults tend to maintain both target and distracting items. Similarly, evidence of age-related reduced suppression of the default mode network (Raichle et al., 2001) during task execution has been linked to a deficit in cognitive control, which hampers efficient resource allocation to task-related areas with a consequent negative impact on WM performance in the elderly (Sambataro et al., 2010). Finally, the idea of an alternative use of processing resources would also be in line with the Compensation-Related Utilization of Neural Circuits Hypothesis (CRUNCH; Reuter-Lorenz and Cappell, 2008). CRUNCH states that, compared to younger adults, elderly recruit more neural resources (and exhaust them) at lower loads and are left without additional cognitive supplies when task demands further increase. A practical example of age-related qualitative changes in resource allocation might come from studies on the Posterior-Anterior Shift in Aging (PASA; Davis et al., 2008) research line: Elderly show increased engagement of frontal areas that correlates with reduced activity of posterior regions. Such activation pattern was suggested to reflect the involvement of high-order cognitive processes in response to deficits of posterior brain areas.

Some final considerations need to be addressed. Given that we focused our mini-review on the WM domain, our conclusions might not be generalized to other cognitive domains, even though age-related limitations in WM seem to account for age-related differences across different tasks (including memory – Baudouin et al., 2009 – and non-memory related domains – Van der Linden et al., 1999; Chen and Li, 2007; Borella et al., 2011). Moreover, while it would be desirable to obtain measures of general cognitive functioning to correlate with neural activations (e.g., Wiegand et al., 2018), such a unique and exhaustive performance index is not easy to choose or compute.

To conclude, on the basis of the recent findings discussed in this mini-review, we suggest that neural measures represent a powerful tool when investigating age-related differences in cognitive resource deployment, provided that some confounding factors are taken into account. Moreover, according to our view, a qualitative change in the deployment of cognitive resources instead or along with a quantitative reduction in the pool of available resources is an alternative hypothesis that deserves further consideration.

## Author Contributions

CT and VM equally contributed to conceptualization, literature overview, and writing of the manuscript. All authors contributed to the article and approved the submitted version.

## Funding

The publication of this article was funded by the Reversing Age and Resilience in the Elderly (RARE-net) project, University of Trento. CT is supported by a research fellowship from the Fondazione Cassa Di Risparmio Di Trento e Rovereto (CARITRO).

## Conflict of Interest

The authors declare that the research was conducted in the absence of any commercial or financial relationships that could be construed as a potential conflict of interest.

## Publisher’s Note

All claims expressed in this article are solely those of the authors and do not necessarily represent those of their affiliated organizations, or those of the publisher, the editors and the reviewers. Any product that may be evaluated in this article, or claim that may be made by its manufacturer, is not guaranteed or endorsed by the publisher.
